# A humanized *Caenorhabditis elegans* model for studying pathogenic mutations in VPS45, a protein essential for membrane trafficking, associated with severe congenital neutropenia

**DOI:** 10.17912/micropub.biology.001052

**Published:** 2023-11-27

**Authors:** Keiko Gengyo-Ando, Minoru Tateyama, Shohei Mitani, Hideki Ando, Junichi Nakai

**Affiliations:** 1 Oral Physiology, Tohoku University Graduate School of Dentistry, Miyagi, Japan; 2 Physiology, Tokyo Women's Medical University, Tokyo, Japan

## Abstract

VPS45, one of the essential membrane trafficking factors, has been identified as a cause of severe congenital neutropenia 5 (SCN5), but its pathophysiological role remains unknown. Here, we developed a humanized *C. elegans* model for three pathogenic VPS45 variants. We found that wild-type human VPS45 functionally complemented the loss of *C. elegans* VPS-45 , and the pathogenic human VPS45 variants functioned almost normally with respect to larval development and endocytosis in *C. elegans* . These results suggest that SCN5-associated mutations have little effect on the core function of VPS45, and/or that the degree of VPS45 requirement varies, depending on the cell/tissue.

**Figure 1. Functional analysis of pathogenic VPS45 variants in C. elegans:**
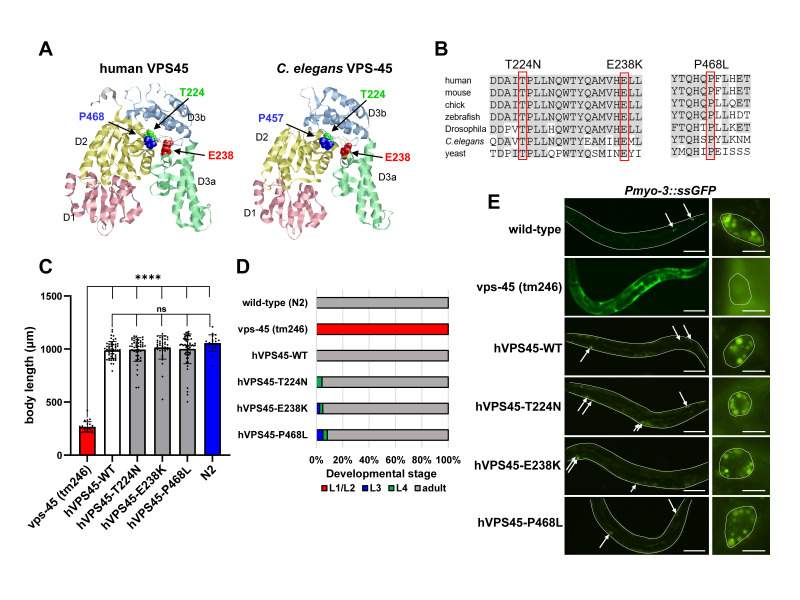
(A) Structures of human VPS45 and *C. elegans* VPS-45 proteins based on I-TASSER structural modeling. The 3D models with the highest confidence score are shown. The VPS45 proteins contain three domains, with domain 3 split into D3a and D3b. The SCN5-associated missense mutations are clustered in the putative hinge region. (B) Alignment of VPS45 protein sequences from Homo sapiens (NP_009190), Mus musculus (NP_038869.1), Gallus gallus (XP_040508302.1), Danio rerio (NP_001243585.1), Drosophila melanogaster (AAF54403.1), Caenorhabditis elegans (NP_741714.1), Saccharomyces cerevisiae (CAA96801.1). The SCN5-associated residues are boxed, and the amino acid residues conserved among the VPS45 homologs are highlighted in gray. (C) Rescue experiments of SCN5-associated hVPS45 mutants for *ts* larval lethality. Bar graphs and dots represent mean and individual body lengths of animals cultured at the restrictive temperature (25℃), respectively. Error bars indicate SEM. A total of 40-60 animals from two independent lines were measured for each hVPS45 construct (WT or variant). **** *p* <0.0001, ns:not significant. (D) Proportions of developmental stages of animals measured in (C). (E) Fluorescence micrographs of adult hermaphrodites of wild-type, *vps-45 (tm246)* , and hVPS45 strains (WT and variant) carrying the *arIs37 [myo-3p::ssGFP]* . The left column shows worms at low magnification and the right column shows individual coelomocytes at high magnification. In the *vps-45 (tm246)* mutant, the secreted GFP is not endocytosed in the coelomocytes and accumulates in the body cavity. The endocytosis defects were rescued by expression of hVPS45 (wild-type or variant). Arrows indicate coelomocytes endocytosing GFP. Individual worms and coelomocytes are indicated by white lines. Scale bars, 100 µm (left panels) and 10 µm (right panels).

## Description

Severe congenital neutropenia (SCN) is a primary immunodeficiency disorder, which is genetically heterogeneous and characterized by the decreased number and dysfunction of neutrophils, suffering from early onset of severe bacterial and fungal infections (Skokowa et al., 2017; Spoor et al., 2019) . In 2013, a homozygous missense variant in VPS45 gene, which encodes a protein essential for membrane trafficking, was first reported as a cause of a novel syndromic form of SCN (SCN5), characterized by progressive bone marrow fibrosis, organomegaly, and osteosclerosis (Stepensky et al., 2013; Vilboux et al., 2013) .

VPS45 belongs to the Sec1/Munc18 (SM) family of proteins and regulates SNARE-mediated membrane fusion in the endosomal/lysosomal system in yeast, *C. elegans* , *Drosophila* , zebrafish, and mammals (Bryant et al 1998; Nielsen et al 2000; Gengyo-Ando et al., 2007; Morrison et al., 2008; Mochizuki et al., 2018) . To date, three homozygous VPS45 missense variants (T224N, E238K, and P468L) have been identified in SCN5 patients (Stepensky et al., 2013; Vilboux et al., 2013; Meerschaut et al., 2015; Shah et al., 2017; Shadur et al., 2018) . The patient-derived cells (neutrophils and fibroblasts) with these mutations exhibit impaired cell migration and increased apoptosis. A recent study showing embryonic lethality in VPS45 knockout mice (Frey et al., 2021) suggests that the patient-associated VPS45 variants are at least partially functional *in vivo.* However, the functional significance and pathological role of the VPS45 variants remain largely unknown.

*vps-45 ( tm246 )* , a null mutant of *C. elegans* VPS45 homologue, exhibits endocytic defects and *ts* (temperature-sensitive) lethal phenotype. We previously showed that mouse VPS45 can rescue *ts* lethality in *vps-45 ( tm246 )* , whereas yeast VPS45 cannot (Gengyo-Ando et al., 2007) . This suggests that the function of VPS45 in membrane trafficking is shared among animals. The aim of our research is to develop an animal model to assess the physiological role of disease-associated variants of VPS45 in a rapid and cost-effective manner. Here, we created and analyzed *C. elegans* models by substituting endogenous VPS45 protein ( VPS-45 ) with human VPS45 protein (hVPS45) (wild-type or SCN5-associated variant forms).

The SM family proteins are composed of four subfamilies (Sec1, Sly1, VPS33, and VPS45) and share a conserved topology with three domains (domain 1, domain 2, domain 3a, and 3b) (Archbold et al., 2014) . So far, no crystal structure has been determined for animal VPS45. To study the structure and function of VPS45, we first performed an *in silico* structure prediction of human and *C. elegans* VPS45 ( Fig. 1A ). We constructed 3D models using the I-TASSER server, an automated protein structure and function prediction platform (Roy et al., 2010; Zhang 2008) (Fig.1A). The structure of *C. elegans* VPS-45 was very similar not only to that of human VPS45 ( Fig. 1A ), but also to that of other SM proteins whose crystal structure was analyzed (Bracher et al., 2000; Misura et al., 2000; Bracher and Weissenhorn 2002; Baker et al., 2013; Eisemann et al *.* , 2020). That is, like other SM proteins, *C. elegans* VPS-45 had a conserved topology of a three-domain structure with a central cleft between domains 1 and 3a. All three wild-type residues (T224, E238, P468), which are mutated in SCN5, are conserved in *C. elegans* and other species (Fig.1B). They are clustered in a hinge region of VPS-45 (Fig.1A). These structural features are consistent with structural modeling studies of hVPS45 (Shah et al., 2017) .

Next, to examine the functional effects of the SCN5-associated VPS45 variants *in vivo* , we conducted knockout-and-rescue approach. The lethal phenotype of *vps-45 ( tm246 )* can be rescued by a VPS-45 minigene, which expresses *C. elegans* VPS-45 under the ubiquitous *eft-3* promoter (Kage-Nakadai et al *.* , 2012). Therefore, we generated transgenic lines carrying hVPS45 minigenes that express wild-type (WT) or variant (T224N, E238K, P468L) hVPS45 under the *eft-3* promoter in the *vps-45* *( tm246 )* null mutant background. First, we tested whether the *ts* lethality of *vps-45* null mutants could be rescued by hVPS45-WT. The hVPS45-WT transgenic strains and control strains were grown at the permissive temperature (15°C). Newly hatched L1 larvae (0-4 hours after hatching) were collected and incubated for 48 hours at the restrictive temperature (25°C). In this condition, *vps-45 ( tm246 )* arrested at the early larval stage (L1/L2) with complete penetrance, while the humanized transgenic animals expressing hVPS45-WT developed into adult worms comparable in length to the wild-type N2 strain [ *vps-45 ( tm246 )* , 267.4±9.9 µm, n=24; N2 , 1057.1±17.4 µm, n=20; hVPS45-WT, 989.6±11.2 µm, n=52] ( Fig. 1C ). This indicates that the human VPS45 protein has a function that fully rescues the lethal phenotype of *C. elegans* VPS-45 depletion. We then analyzed the functional effects of the three SCN5-associated hVPS45 missense variants on growth. We found that these SCN5-associated variants had little effect on the rescue ability of hVPS45 protein; most animals grew to adulthood with lengths comparable to hVPS45-WT and N2 controls (hVPS45-T224N, 996.2±16.2 µm, n=47; hVPS45-E238K, 1015.6±17.2 µm, n=41; hVPS45-P468L, 1001.9±18.1 µm, n=60). Notably, some hVPS45 mutant worms showed a slight delay in their development as compared to the control (hVPS45-WT, adult 100%, n=52; hVPS45-T224N, adult 95.7%, n=47; hVPS45-E238K, adult 95.2%, n=41; hVPS45-P468L, adult 91.7%, n=60). *p* values of hVPS45-T224N, -E238K, and -P468L to hVPS45-WT were p=0.066, p=0.053, and p<0.05, respectively.

Next, we examined the functional effects of hVPS45 variants on endocytosis. *C. elegans* has scavenger cells called coelomocytes in the pseudocoelom. These cells take up a marker for fluid-phase endocytosis, such as a secreted form of GFP (ssGFP) from muscle cells (Fares and Greenwald, 2001) . *vps-45 ( tm246 )* mutants exhibit a Coelomocyte uptake-defective (Cup) phenotype at 20℃; inability to endocytose secreted GFP, resulting in accumulation of GFP in the pseudocoelom (Gengyo-Ando et al *.* , 2007) (Fig.1E). As expected from the above rescue experiments, the SCN mutant strains were found to restore the endocytosis defect almost indistinguishably from the wild type (Fig.1E).

In summary, we developed an efficient and economical animal model for pathological analysis of the human genetic disease SCN5 *.* The human and *C. elegans* VPS45 are structurally similar and human VPS45 can functionally complement *C. elegans* VPS45 depletion. Human VPS45 with SCN5-associated missense mutations functions normally in terms of larval development and endocytosis, except for a slight delay in development. This implies that these variants are simply weak alleles and/or that the degree of VPS45 requirement could vary depending on the cell/tissue, while overexpression from a high copy array with a strong promoter ( eft-3 p) might mask the reduced ability of the VPS45 mutants and their differences in functionality. The humanized worm model we developed here would provide a useful platform with which to analyze detailed functions of pathogenic VPS45 variants *in vivo* .

## Methods

***C. elegans* strains and maintenance:** *C. elegans* strains were cultured using standard techniques (Brenner 1974) . The wild-type strain Bristol N2 and *arIs37 [myo-3p::ssGFP + dpy-20 (+)]* were obtained from the Caenorhabditis Genetics Center. *vps-45 ( tm246 )* and *vps-45 ( tm246 ); arIs37* were maintained at 15℃ as previously described (Gengyo-Ando et al., 2007) . The strains generated in this study are shown in Reagents.

**Plasmid construction:** The cDNA sequence encoding human VPS45 (hVPS45-WT) was amplified from a U937 cDNA library using primers hvps45#F1: 5'-TGCGGCCGCATGAACGTGGTTTTTGCTGTG-3' and hvps45#R1: 5'-TAGATCTTCATCTTCTGCTTGCTGACC-3' and cloned into pGEMTeasy (Promega). The cDNAs encoding hVPS45-T224N, hVPS45-E238K, and hVPS45-P468L were generated using the KOD-plus mutagenesis kit (Toyobo). Primers used for mutagenesis are as follows; hvps45_T224N#F1: 5'-AaCCCATTGCTAAACCAGTGGAC-3', hvps45_T224N#R1: 5'-GATGGCATCATCACAGCGAT-3'; hvps45_E238K#F1: 5'-aAACTACTAGGCATAAACAACAATC-3', hVPS45_E238K#R1: 5'- GTGGACCATGGCCTGATATG-3', hvps45_P468L#F1: 5'- CtTTTCCTACATGAAACCCTGGA-3', hVPS45_P468L#R1: 5'- TTGATGCTGTGTATATACATTTTC-3'. To generate hVPS45 minigenes, EGFP sequence in a pFX_EGFPT vector (Gengyo-Ando et al., 2006) containing the *eft-3* promoter was removed by NotI/BglII digestion and then replaced with the NotI/BglII fragments containing wild-type or mutant hVPS45. All constructs were verified by DNA sequencing.

**Generation of humanized strains:** To generate transgenic animals, DNA construct encoding hVPS45 (wild-type or variant) was co-injected into the gonads of N2 hermaphrodites at 20 ng/µl each, along with *gcy-10p::RFP* at 180 ng/µl as an injection marker, using a standard protocol (Mello et al., 1991) . Stable transgenic lines carrying extrachromosomal arrays were obtained at the F2 generation and maintained. To generate humanized strains, two independent lines for each hVPS45 construct were crossed into *vps-45 ( tm246 )* , and then *vps-45 ( tm246 )* homozygous mutants carrying the hVPS45 transgenes were obtained ( QJ4126 - QJ4133 ). The *tm246* allele of each humanized strain was confirmed by PCR amplification using primers that span the deletion in *vps-45 ( tm246 )* (Gengyo-Ando et al., 2007)

**Body length:** Animals were observed and photographed under an SZX16 stereomicroscope (Olympus) equipped with a DP28 digital camera (Olympus). The cellSens software (version 1.7, Olympus) was used to measure the length of the worm body. The developmental stage of each animal was assessed by worm length and vulval morphology as previously described (Porta-de-la-Riva et al., 2012) . In the humanized strains, RFP-positive worms carrying transgenes were examined.

**Endocytosis assay:** The strain *arIs37 [myo-3p::ssGFP, dpy-20 (+)]* constitutively expresses ssGFP, which is secreted from muscle cells into the body cavity (Fares and Greenwald, 2001) . QJ4134 , QJ4135 , QJ4136 , or QJ4137 were obtained by crossing QJ4126 , QJ4128 , QJ4130 , or QJ4132 into the strain *arIs37* , respectively. The resulting hVPS45 strains carrying *arIs37* were cultured at 20°C. *vps-45 ( tm246 ); arIs37* was incubated at 20 °C for 24 hours prior to assay *.* Adult worms were mounted on agarose pads and viewed on a fluorescence microscope (ECLIPSE Ni, Nikon), using a 10× objective (CFI Plan Apo Lambda, 0.45 NA, Nikon) or 100x objective (CFI Plan Apo Lambda, 1.45 NA, Nikon). Images were taken with DP28 digital camera (Olympus). Animals were immobilized with 5 mM levamisole.

**Statistics:** One-way ANOVA followed by Dunnett's test was performed using GraphPad Prism 10.0.3 (GraphPad Software) ( Fig. 1C ). Hypothesis testing for difference in population proportions was performed using Excel Tokei (Social Survey Research Information) ( Fig. 1D ).

**3D model:** Prediction of 3D structure of *C. elegans* VPS-45 and human VPS45 were performed using the I-TASSER server (https://zhanggroup.org/I-TASSER/) (Zhang 2008; Roy et al., 2010) . The domain structures of *C. elegans* VPS-45 and human VPS45 were based on the three-domain structure of SM proteins defined by Misura et al (Misura et al., 2000) . Structural representation was performed using RasMol (version 2.7.5.2), a program for molecular graphics visualization (http://www.openrasmol.org/).

## Reagents

**Table:** 

Strain	Genotype	Source
QJ4126	*vps-45 ( tm246 )X; jqEx611 [ eft-3 p::hvps45(WT)+gcy-10p::RFP] *	This work
QJ4127	*vps-45 ( tm246 )X; jqEx613 [ eft-3 p::hvps45(WT)+gcy-10p::RFP] *	This work
QJ4128	*vps-45 ( tm246 )X; jqEx618 [ eft-3 p::hvps45(E238K)+gcy-10p::RFP] *	This work
QJ4129	*vps-45 ( tm246 )X; jqEx622 [ eft-3 p::hvps45(E238K)+gcy-10p::RFP] *	This work
QJ4130	*vps-45 ( tm246 )X; jqEx625 [ eft-3 p::hvps45(T224N)+gcy-10p::RFP] *	This work
QJ4131	*vps-45 ( tm246 )X; jqEx626 [ eft-3 p::hvps45(T224N)+gcy-10p::RFP] *	This work
QJ4132	*vps-45 ( tm246 )X; jqEx628 [ eft-3 p::hvps45(P468L)+gcy-10p::RFP] *	This work
QJ4133	*vps-45 ( tm246 )X; jqEx629 [ eft-3 p::hvps45(P468L)+gcy-10p::RFP] *	This work
QJ4134	*vps-45 ( tm246 )X; arIs37 [myo-3p::ssGFP+ dpy-20 (+)]; jqEx611 [ eft-3 p::hvps45(WT)+gcy-10p::RFP] *	This work
QJ4135	*vps-45 ( tm246 )X; arIs37 [myo-3p::ssGFP+ dpy-20 (+)]; jqEx618 [ eft-3 p::hvps45(E238K)+gcy-10p::RFP] *	This work
QJ4136	*vps-45 ( tm246 )X; arIs37 [myo-3p::ssGFP+ dpy-20 (+)]; jqEx625 [ eft-3 p::hvps45(T224N)+gcy-10p::RFP] *	This work
QJ4137	*vps-45 ( tm246 )X; arIs37 [myo-3p::ssGFP + dpy-20 (+)]; jqEx628 [ eft-3 p::hvps45 (P468L) + gcy-10p::RFP] *	This work
